# The Coronary Venous System in Acute Coronary Syndrome: A Narrative Review

**DOI:** 10.3390/biomedicines14051063

**Published:** 2026-05-07

**Authors:** Ercan Akşit, Cengiz Demir, Uğur Özpınar, Esra Duman Acar

**Affiliations:** 1 Department of Cardiology, Faculty of Medicine, Çanakkale Onsekiz Mart University, 17020 Canakkale, Turkey; esiraduman@gmail.com; 2 Department of Cardiology, Muğla Menteşe State Hospital, 48000 Mugla, Turkey; krdcengizdemir@gmail.com; 3 Department of Cardiology, Biga State Hospital, 17200 Canakkale, Turkey; drugurozpinar@gmail.com

**Keywords:** acute coronary syndrome, coronary venous system, coronary sinus thrombosis, myocardial venous bridge, persistent left superior vena cava

## Abstract

Acute coronary syndrome (ACS) remains a leading cause of death and morbidity worldwide. The pathophysiology of ACS has been largely interpreted through abnormalities of the coronary arteries and the microvascular bed. However, the coronary circulation is fundamentally a closed-loop system, in which the venous component represents the final link in myocardial blood return. In contrast to the extensive literature on arterial and microvascular disease, there are relatively few studies on the coronary venous system (CVS) in the context of ACS. The CVS is important in relation to ACS from two complementary perspectives. First, structural or functional abnormalities of the CVS can contribute to myocardial ischemia; second, coronary venous flow can reflect the hemodynamic outcomes of acute ischemia and reperfusion. The ‘vascular waterfall’ phenomenon is considered one of the primary mechanisms governing coronary venous return, linking myocardial compression and venous pressure to the flow from the coronary sinus (CS) into the right atrium. Experimental and clinical evidence has shown that CS thrombosis is associated with myocardial infarction and may also complicate ACS. Furthermore, studies evaluating CS blood flow generally show a decrease in the acute phase of ischemia and an increase after reperfusion. However, the existing evidence is limited and largely based on small observational studies. Therefore, this review aimed to examine the pathophysiological mechanisms and hemodynamic behavior of the CVS in ACS, starting from embryological development.

## 1. Introduction

In its broadest sense, coronary artery disease (CAD) remains the leading cause of death worldwide [1]. Acute coronary syndrome (ACS), a critical manifestation of CAD, is life-threatening because it begins suddenly and unpredictably [2]. Health expenditures are increasing worldwide in parallel with the rising incidence of cardiovascular disease (CVD) [3,4]. Among all CVDs, the economic burden of CAD is significantly increasing, with the highest cost being hospital admissions related to ACS [4]. In the United States alone, the economic burden of CVD is expected to increase from USD 393 billion in 2020 to 1490 billion by 2050 [5]. While CAD was traditionally considered a disease primarily affecting the elderly, it has become increasingly prevalent in younger and middle-aged individuals in recent years [6,7]. Importantly, this trend appears to be gender-specific, with recent evidence suggesting that the increasing burden of ACS in younger populations disproportionately affects women, particularly those presenting with myocardial infarction with non-obstructive coronary arteries (MINOCA). This observation closely aligns with the growing recognition of MINOCA and Type 2 myocardial infarction (T2MI) [8]. This trend has prompted increasing research into its underlying mechanisms and management [9,10,11,12].

Plaque rupture, embolism, thrombosis, and dissection generally play roles in the pathophysiology of ACS [13]. In CAD and ACS, research and treatment goals are focused on the coronary arteries and the microvascular bed [14]. The coronary blood flow is a closed circuit, originating from the coronary arteries, continuing through the microvascular bed, and then through the coronary venous system (CVS), with the force that enables this circulation explained by the ‘vascular waterfall’ phenomenon [15]. Although the coronary sinus (CS) is commonly used as an anatomical access point in electrophysiology and interventional cardiology, its role in CAD etiology has received limited attention. The CS has been utilized in ACS, both during percutaneous coronary intervention (PCI) and in coronary bypass graft (CABG) surgery, to increase retrograde perfusion or as a therapeutic modality to reduce refractory angina in chronic coronary syndromes [16,17,18]. In particular, one of the rare causes of MINOCA—a subtype of ACS—may be due to underlying pathologies in the CVS.

### Aims of This Review

This review aims to better understand this issue by starting from the embryonic process of the CVS and, given the information in the literature, investigating both its place in the etiology of ACS and its hemodynamic behavior in the acute and chronic phases.

## 2. Embryology and Histology of the Coronary Venous System

### 2.1. Embryologic Development

The importance of this subheading is underscored by the following question: just as coronary artery anomalies can cause ACS by leading to a critical disruption in the myocardial supply–demand balance, could CVS anomalies also cause such a disruption [19,20,21,22]?

The CS develops from the left common cardinal vein and the embryonic sinus venosus; simultaneously, other major cardiac veins develop in a process involving remodeling of the primitive subepicardial vascular plexus. The vena cardiaca magna, vena cardiaca parva, and vena cardiaca media drain into the CS, while the vena cardiaca anteriores open directly into the right atrium [23,24]. The precise embryological origin of the Thebesian veins (venae cordis minimae) remains less understood, compared with that of the major veins. The most prominent theory is that this system originates embryologically from an intertrabecular network, distinct from the main venous circulation [24,25]. Epicardial-derived cells (EPDCs) undergo epithelial-to-mesenchymal transition, migrate to the subepicardial space, and subsequently form the vascular plexus. Key molecules involved in this process and subsequent CVS remodeling include vascular endothelial growth factor (VEGF); chicken ovalbumin upstream promoter–transcription factor -II (COUP-TFII); transforming growth factor-beta (TGF-β); platelet-derived growth factor (PDGF); Eph/Ephrin; and Notch Signaling [26,27,28]. The direction in which these signals shift highlights subtle molecular differences between the arterial and venous systems, with the Ephrin-B2 gene selectively expressed in the arterial endothelium; conversely, the Eph-B4 receptor is expressed on the venous endothelial side. Furthermore, dysregulation in Notch activity during embryological development of the coronary vascular circulation can cause arteriovenous malformations [29,30].

### 2.2. Histological and Anatomical Features

The CVS consists of three layers: the innermost tunica intima, the middle tunica media, and the outermost tunica adventitia [31]. The tunica adventitia is the predominant layer of the CVS, composed of dense, irregular connective tissue. This layer contains the vasa vasorum, fibroblasts, collagen, elastic tissue, and autonomic nerves, which provide flexibility and strength to the CVS. The tunica media consists of smooth muscle cells arranged circularly with elastic and collagen fibers. The tunica media is important for venous tone, but is thinner than its arterial counterpart because blood flows at a lower pressure here. The tunica intima comprises a thin subendothelial connective tissue layer and a continuous, monolayer of endothelial cells; this layer maintains a non-thrombogenic interface to optimize laminar flow in an environment that is otherwise prothrombotic due to low-pressure conditions [31,32,33]. The coronary venous wall contains myocardial sleeves. These sleeves extend from the endocardial layer to the tunica adventitia and play a crucial role in the hemodynamics of venous flow [34].

**Figure 1:**
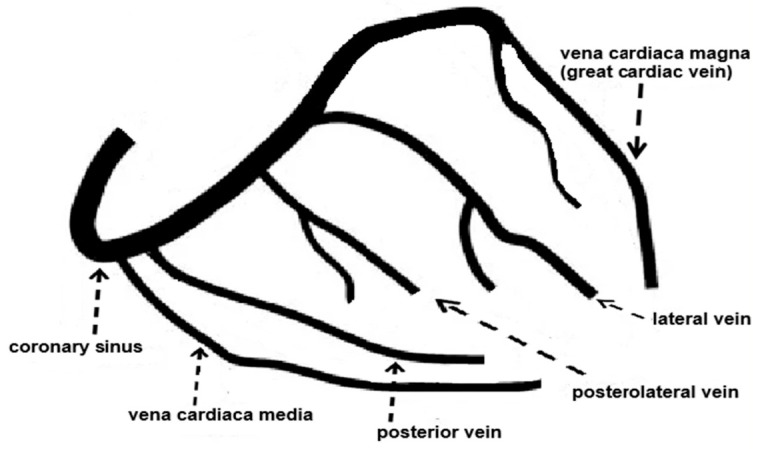
Anatomical structure of the mature coronary venous system.

**Figure 2:**
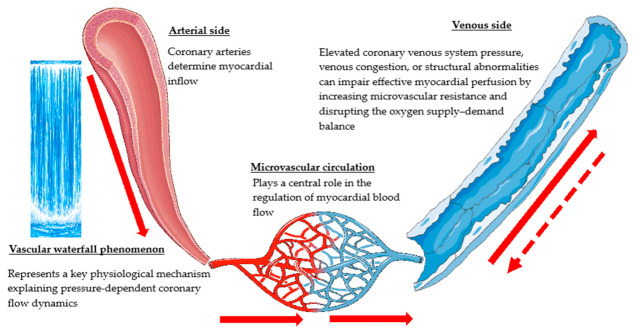
Components of coronary vascular circulation. A schematic showing the closed-loop integration of arterial supply, microvascular beds, and venous drainage. The thick red lines represent the normal antegrade direction of flow, characterized by the ‘vascular waterfall’ phenomenon. The red dashed line indicates coronary venous reflux flow, which may be observed selectively within the venous bed under certain hemodynamic conditions.

A schematic representation of the anatomical shape of a developed CVS is illustrated in Figure 1. When examining this figure, it is important to remember that in conventional coronary angiography (CAG), the coronary arteries are the only ones that are opacified; therefore, the major CVS—which runs parallel to the artery—remains radiographically occult. The coronary vascular circulation is composed of several integral parts, as shown in Figure 2.

Within the smaller tributaries of the CVS—as in the small veins that drain other organs of the body—on average, there are three unnamed valves [35,36]. Of the valves with specific names, the Thebesian valve is situated at the orifice of the CS, while the Vieussens valve is found in the great cardiac vein to prevent retrograde flow [36,37].

### 2.3. Coronary Venous System Anomalies and Their Influence on Coronary Arterial Perfusion

In current diagnostic imaging techniques for ACS, the CVS is overshadowed by the arterial system, highlighting the importance of evaluating CVS anomalies.

*Enlargement of the CS*: This condition can be acquired (e.g., due to pulmonary arterial hypertension, severe heart valve disease, severe heart failure, and atrial fibrillation) or congenital [38,39]. Congenital conditions are examined in two categories, based on the presence or absence of a left-to-right shunt [40,41]. The most common cause of CS enlargement without an L-R shunt is persistent left superior vena cava (PLSVC). Autopsy studies have suggested that the prevalence of PLSVC in the general population is approximately 2% [40]. Left-to-right shunt CS dilation results from an abnormal connection between the CS and the left atrium [41,42,43]. Partial anomalous pulmonary venous return has a prevalence of 0.7% in the general population, while total anomalous pulmonary venous return accounts for approximately 3% of congenital heart defects [43]. Among these anomalies, the most clinically relevant anomaly is the coronary arteriovenous fistula, which originates from persistent intratrabecular sinusoids between the coronary arteries and the CS [44,45,46,47]. The overall incidence of coronary artery fistulas is 0.8% in CAG imaging, while it is detected at a frequency of 0.07% in coronary computed tomography angiography (CCTA). Among all coronary fistulas, coronary artery fistulas terminate in the CS in approximately 2.38% of cases [46].

*Coronary sinus atresia and hypoplasia*: In the presence of this congenital anomaly, coronary venous return cannot occur via the CS; instead, venous return is diverted into the right atrium through the dilated Thebesian veins [48,49,50]. In one study, the frequency of CS orifice atresia in autopsies was reported as 0.03%, while its frequency detected in CCTA was 0.11% [50].

**Figure 3:**
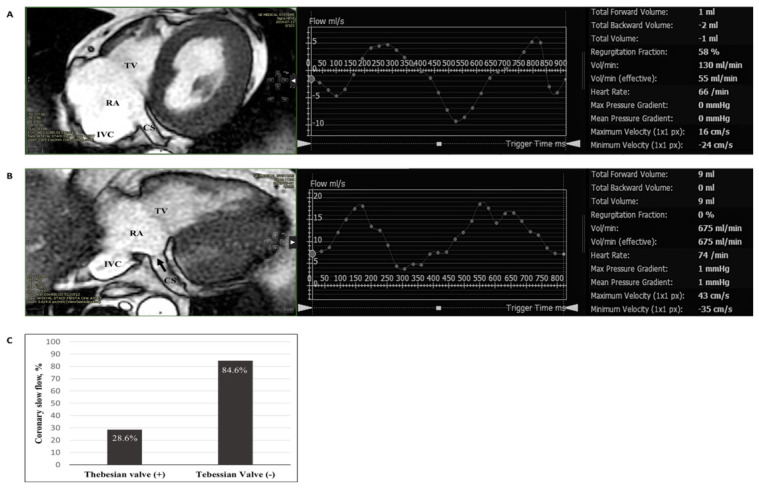
Cardiac magnetic resonance imaging. (**A**) In a patient without a Thebesian valve in the CS, coronary venous regurgitant flow is reflected in the flow–time curve on the left; (**B**) in a patient with a Thebesian valve in the CS (black arrow), the flow–time curve shows anterograde flow, with no regurgitant flow detected; (**C**) only 28.6% of patients with a Thebesian valve in the CS have slow coronary flow. Abbreviations: coronary sinus (CS), inferior vena cava (IVC), right atrium (RA), tricuspid valve (TV). Adapted from our previous study [52].

Beyond these abnormalities, the absence of CVS valves is not considered a congenital abnormality but is present in some individuals; this has recently spurred clinical research regarding this phenomenon’s effects on the heart. Previous studies have emphasized that the valves in the CVS are not primitive remnants but are functional, preventing blood from flowing backward against gravity [51]. Previous studies have reported a variable prevalence of CVS valves. The Thebesian valves and Vieussens valves are present in 75% and 80% of individuals. Moreover, valves are observed in 60% of middle cardiac veins and 30% of left posterior veins and left marginal veins, but only 12% of the anterior cardiac veins possess these structures [36]. CS reflux and coronary slow flow may be related in this closed circulation, and coronary slow flow has been observed more frequently in patients without a Thebesian valve (Figure 3) [52].

**Figure 4:**
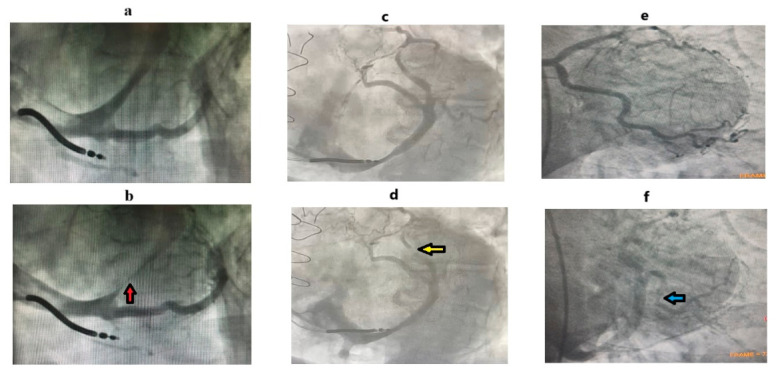
Coronary venography and conventional coronary angiography images. (**a**) Venography shows that the CS and its branches appear to be normal in the diastolic phase. (**b**) MVB causes severe stenosis in the diastolic phase (red arrow). This finding should be considered when the CS catheter is not positioned in that branch. (**c**) Distal branches of the CS appear normal during the diastolic phase. (**d**) MVB in the distal branch of the CS (yellow arrow) becomes prominent in the systolic phase. (**e**) Coronary artery system of a patient undergoing invasive coronary angiography due to ACS. (**f**) The image shows an incidental MVB in the left marginal vein (blue arrow). While the compression caused by the muscular bridge in the artery is minimal, a significant compression can be observed in the left marginal vein, which runs parallel to the artery. As we discussed in the histology section of this review, this may be due to the thinner and more compliant wall structure of venous tunica media compared to the artery, and its greater susceptibility to compression from the myocardial bridge. Abbreviations: acute coronary syndrome (ACS), coronary sinus (CS), myocardial venous bridge (MVB). Adapted from our previous study [57].

*Myocardial bridge* is a congenital anomaly of the coronary arteries and involves the partial or complete course of the artery through the myocardium. Myocardial bridges have also been shown to cause ACS and accelerate and predispose patients to atherosclerosis [53,54,55,56]. Conversely, myocardial venous bridges (MVBs) have only been detected in cadaver studies; however, they have recently been observed incidentally during venography prior to implementing cardiac resynchronization therapy leads and during conventional CAG (Figure 4) [57]. Anatomically and histologically, two forms of the myocardial bridge have been categorized: the first involves only the coronary artery as its intramyocardial segment, and the concomitant involvement of both the artery and the coronary vein [58]. According to one autopsy study, in myocardial bridges, both the artery and the vein run together within the myocardial muscle, accounting for 3.5% of cases [58].

**Figure 5:**
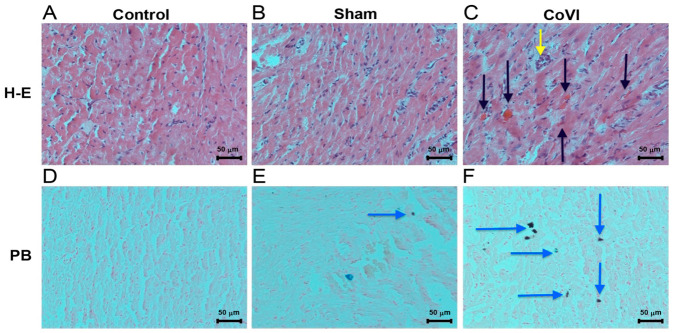
Representative histopathological sections of experimental preparations for a coronary venous insufficiency (CoVI) model: Hematoxylin–eosin (HE) (**A**–**C**) and Prussian blue (PB) (**D**–**F**). Coronary venous insufficiency (CoVI) experimental model showing marked erythrocyte extravasation (black arrow), extravascular macrophage infiltration (yellow arrow), and hemosiderin deposits (blue arrow). Adapted from our previous study [59].

**Figure 6:**
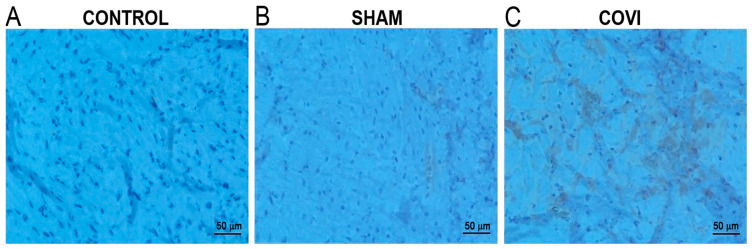
Representative immunohistochemical staining of the experimental preparations for a coronary venous insufficiency (CoVI) model: Matrix metalloproteinase-2 staining was significantly observed in the CoVI group (**C**) compared with the control (**A**) and sham (**B**) groups. Adapted from our previous study [59].

**Figure 7:**
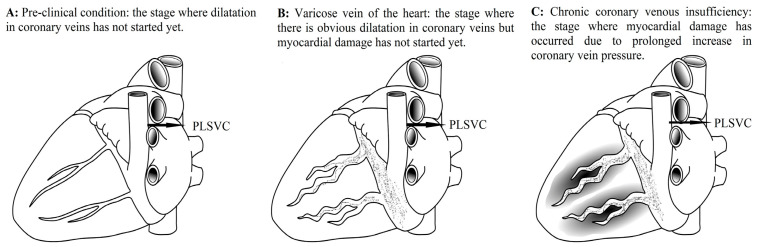
The development process of chronic coronary venous insufficiency as a result of prolonged exposure to venous pressure in a clinical condition such as persistent left superior vena cava (PLSVC), in light of data from the literature. Adapted from our previous study [63].

The potential implications of this pressure elevation within the CVS have been extensively investigated in previous studies. In a previous experimental study, an elevated intraluminal pressure in the CVS and pathophysiological changes similar to those in peripheral chronic venous insufficiency were observed in the myocardial tissue (Figure 5 and Figure 6) [59]. This inseparable connection between the arterial and venous systems is best illustrated in the chronic venous insufficiency of the lower extremity where when one system is impaired, the other is also affected. Studies have shown that disease in these two structures is not just concomitant; rather, chronic venous insufficiency is also directly related to the severity of peripheral artery disease [60,61,62]. This histopathological process and the remodeling it can cause in myocardial tissue during the chronic phase may explain why PLSVC is not only an incidentally detected pathology, but can also cause certain arrhythmias, heart failure, and MINOCA (Figure 7) [63,64,65,66,67,68]. A detailed appraisal of current CVS research is essential to elucidate the role of coronary veins in the etiology of ACS, particularly regarding the pathophysiology of MINOCA and its subsequent clinical outcomes.

## 3. Hemodynamics of the Coronary Venous System in ACS

### 3.1. Dynamics of Blood Flow in the Coronary Venous System

It is assumed that the primary force driving coronary venous flow is the vascular waterfall phenomenon [15]. This phenomenon highlights the initial flow of blood from the coronary arteries at a certain pressure, while the final determinant of its return is the pressure difference between the coronary veins and the right atrium. This pressure difference is affected by three factors: myocardial systolic compression, myocardial diastolic relaxation, and basal right atrial pressure [69,70,71,72]. In the vascular waterfall phenomenon, the primary determinant of flow depends on the magnitude of the difference between arterial pressure and tissue pressure; in other words, the flow in a waterfall is determined by the driving force (arterial pressure) and the maximum downstream force (tissue and the right atrium pressure) that the driving force must overcome. An extension of the vascular waterfall concept is the heart’s intramyocardial pump function. This intramyocardial pumping action of the heart explains the increases in coronary venous outflow observed during ventricular systole [73]. CVS blood flow is affected by sympathetic and parasympathetic innervation; among the mediators, nitric oxide is the most important vasodilator, while endothelin-1 is the most important vasoconstrictor of this system [74,75,76,77]. Three main drugs also positively affect blood flow by acting on the CVS, as follows: beta-blockers reduce venocontraction caused by sympathetic innervation; nitrates reduce wall stress by markedly dilating the venous vessel; and diuretics reduce this stress by preventing coronary venous congestion [78,79,80].

Vascular tone in the coronary arteriolar and venous systems is affected by intravascular pressure. The two following fundamental mechanisms protect both systems from endothelial damage: firstly, contraction of microvascular smooth muscle in response to increased pressure, and secondly, vasodilation of vascular smooth muscle in response to decreases in blood pressure [71,75,78].

A comprehensive understanding of these mechanisms allows for a clearer evaluation of how CVS pathologies contribute to ACS, as well as how coronary veins respond to conventional arterial and microvascular disease.

### 3.2. Coronary Venous System and MINOCA

MINOCA represents a multifactorial clinical picture involving multiple underlying mechanisms. Of these, T2MI is defined by an imbalance between myocardial oxygen supply and demand in the absence of acute atheroma plaque rupture; this constitutes a particularly important pathophysiological subcategory [8]. In this context, the various CVS abnormalities discussed in this review may be more appropriately interpreted within the framework of T2MI, rather than classical T1MI.

CVS abnormalities may contribute to MINOCA through two main mechanisms. The first of these mechanisms involves pathologies including PLSVC, CS atresia or hypoplasia, abnormal CS reflux, and MVB, which can lead to increased venous pressure, impaired myocardial drainage, and secondary ischemia. Secondly, the coronary steal phenomenon, particularly seen in coronary arteriovenous fistulas draining into the CS, can reduce effective myocardial perfusion despite angiographically normal coronary arteries.

In an experimental study, Miyahara et al. were among the first to realize that myocardial infarction can occur without significant coronary artery stenosis or vasospasm and that one of the underlying causes may involve the CVS. They injected thrombin into the anterior interventricular vein of their subjects and subsequently observed ST-T changes on electrocardiography and elevated cardiac markers, and no pathology was detected in the coronary arteries during CAG [81]. Wells et al. detected CS thrombosis in a patient diagnosed with spina bifida who had undergone ventriculoatrial shunt revision. Autopsy revealed a secondary myocardial infarction in the region near the conduction system within the interventricular septum, and they stated that this led to the sudden death of the patient [82]. Yeo et al. reported that a thrombus they observed in the CS during catheter ablation caused ST elevation in the inferior leads, reduced left ventricular ejection fraction (LVEF) to 30%, and elevated cardiac markers. They demonstrated that, after successful thrombus aspiration and anticoagulant therapy, cardiac markers regressed and LVEF increased to 60% in the following days [83]. In addition, the literature describes several pathologies that cause CS thrombosis, such as malignancy and autoimmune diseases, but more extensive research is needed to understand how many of these cause MINOCA [84]. In case presentations of patients with PLSVC, this suggests a possible association with CAD [85,86,87]; however, comprehensive studies are needed to understand whether PLSVC plays a role in the pathophysiology of CAD progression in such cases.

A recent case report described an intramyocardial dissecting hematoma associated with a CS diverticulum in a patient with elevated troponin levels but normal coronary arteries on CAG [88]. In this patient, the CS diverticulum was not detected on transthoracic echocardiography (TTE) and was not initially noticeable on cardiac magnetic resonance (CMR) imaging; it was only detected on cardiac CT, which provided the most valuable information. Interestingly, a negative delta wave was detected in lead II. Similarly, a previous study found that a negative delta wave in lead II predicted CS diverticulum with 77% sensitivity [89].

It is well established that coronary artery fistulas cause MINOCA [90,91]. Of the coronary arteriovenous fistulas, those draining into the right atrium are associated with MINOCA, while case reports where they terminate in the CS are associated with chest pain complaints [91,92,93]. As these fistulas are often not detectable on TTE, cardiac CT or CAG is useful in diagnosis in suspected cases. Furthermore, intervention should be considered in the presence of a large fistula, ventricular dysfunction, ischemia, and pulmonary hypertension [94].

MVBs have been demonstrated in recent years using CAG [57]. In a patient with ACS, an MVB (in the left marginal vein) in the venous phase was incidentally detected while investigating collateral branches to the right coronary artery (Figure 4). The presence of myocardial bridges in other coronary venous branches in this patient could not be investigated for ethical reasons, as coronary venous examination in CAG involves more contrasting agents and greater radiation exposure. These patients are difficult to diagnose with conventional diagnostic tools. It is important to remember that if a myocardial bridge causing significant stenosis is detected, both the coronary artery and the vein can be compressed within the myocardium in these patients [58], so cardiac CT or CAG imaging may be necessary in the venous phase as well. Given that this can affect the surgical treatment option in myocardial bridge patients who have developed severe stenosis, treatment strategies for myocardial bridges are not yet well-defined enough to be included in clinical guidelines. Stent implantation is controversial due to the increased risk of coronary arterial perforation, stent fracture, and thrombosis [56,95]. Since stents are implanted in the coronary arteries, they are not effective in treating MVBs. In surgical management, saphenous vein grafts are preferred in CABG procedures because the left internal mammary artery may become occluded due to competition with the coronary artery in these patients [56,95]. More favorable outcomes have been observed in patients undergoing myotomy compared to CABG [96]. If future studies confirm that MVBs are associated with adverse cardiac outcomes, myotomy may become the preferred treatment option for bridges involving both coronary arteries and veins.

To date, there has been no systematic review, meta-analysis, or guidelines examining CVS abnormalities that could cause MINOCA; therefore, the precise extent of these CVS mechanisms that can cause MINOCA remains unclear. In clinical practice, multimodal imaging, including TTE, CCTA, CMR imaging, and venography, may be necessary to identify these CVS abnormalities. Furthermore, functional assessment using CS flow measurements can provide additional information about the hemodynamic significance of these findings.

### 3.3. Studies Examining the Coronary Venous System in ACS

The main basis of studies examining the CVS in ACS is CS blood flow (CSBF) measured via the CS pathway. Most of these studies did not specifically aim to examine the potential role of CVS in ACS [97,98,99,100,101,102,103,104,105,106,107,108,109,110,111,112,113,114,115,116,117,118,119,120,121,122,123,124] and, to date, only one study has demonstrated a relationship between CS reflux and coronary slow flow [52]. Although the number of patients was limited, it is the only clinical study in the literature examining this relationship. Furthermore, coronary slow flow has been found to be less frequent in patients with the Thebesian valve in CS [52]. This study also supports the thesis of Pan et al. that coronary venous valves are functional [51,52].

The aim of most studies, including those involving CVS, was to obtain information about myocardial perfusion in patients with CAD. Assessing myocardial blood flow (MBF) in CAD patients provides important information about the disease prognosis. Although positron emission tomography (PET) is the gold standard method for measuring MBF, its high cost and radiation exposure limit its use [122,123]. Myocardial flow reserve (MFR) is calculated by dividing hyperemic MBF values by resting MBF values, following vasodilator administration [122,123]. Using CMR imaging, it has also been shown that CS blood flow (CSF) correlates with PET-derived MBF [125,126].

In two studies involving patients with left anterior descending artery (LAD) and left main coronary artery (LMCA) lesions, CFR values were calculated using transesophageal echocardiography (TEE). It was found that CFR measured by TEE is a useful diagnostic tool and a significant predictor of LAD and LMCA lesions in these patients [97,98]. In both studies, dipyridamole was used as the agent for the hyperemic phase in CFR measurements. Similarly, it has been shown that measuring CFR values with TEE is a useful method for evaluating diabetic microvascular dysfunction [100]. However, unlike the other two studies conducted with TEE, this study used adenosine triphosphate for hyperemia measurement in CFR analysis. In these studies, the authors stated that they performed the TEE procedure within 1 week following the CAG procedure. Furthermore, the importance of CSF was calculated using TTE, a reproducible, cost-effective, and non-invasive method, and CSF measurement via TTE has also been evaluated in patients undergoing revascularization. In patients who underwent CABG, both preoperative and postoperative evaluations performed within one month showed improvement in CBF and CFR after revascularization [99,102]. In patients who underwent repeat TTE after CABG, a significant increase in CBF was observed at both 1 and 6 months. This study differs from all other studies because it took repeated measurements for CBF [117]. Increased utilization of CMR imaging and larger sample sizes in recent studies have enhanced our understanding, providing more reliable insights into coronary venous hemodynamics. As measured via CMR imaging, CFR has high diagnostic value in predicting decreased fractional flow reserve (FFR) in single-vessel disease, but its value is more limited in multi-vessel disease [107]. Long-term follow-up studies using CMR imaging techniques have shown that CBF and CFR provide complementary prognostic value in predicting cardiovascular events [109,111,112,113]. While other studies have associated low CFR values with MACE, one study has shown an increased risk of MACE with higher CSBF [113]. Although a recent study involving 933 patients with CMR imaging had an average follow-up period of 5.3 years, like all other follow-up studies, patients were contacted through review of medical records and/or telephone interviews [118]. Designing randomized follow-up studies and obtaining serial CS flow measurements from patients could further increase our knowledge on this subject. CS-filling time delay is associated with angina pectoris and MFR reduction in both CCTA and conventional CAG [105,122]. A recent study indicated that there is poor agreement between MFR obtained from PET, the gold standard method, and CFR obtained from CMR imaging, and that they are not interchangeable. Therefore, it emphasized the need for caution when interpreting CFR obtained from CMR in patients with established ischemic CAD [123].

These studies are limited and mostly used a case–control or retrospective design. Most of those reviewed in this article showed that CSBF decreases in ACS and increases after reperfusion. Furthermore, there are no randomized controlled trials on this subject. Future well-designed prospective studies on this topic can be expected to increase our knowledge about the CVS in relation to ACS. A summary of the studies is given in Table 1.

Another point to keep in mind is that CS thrombosis—a rare but potentially fatal complication of ACS—should also be considered [127].

### 3.4. Interventions via the Coronary Venous System in ACS

While diagnostic tools such as conventional CAG, intravascular ultrasound, and FFR are used for the evaluation of coronary arteries, CVS has largely remained inadequately studied in clinical practice. This gap in our diagnostic assessment highlights a significant deficiency in our understanding of total coronary circulation and supports the need to integrate CVS evaluation into contemporary approaches [128]. Although PCI improves clinical outcomes in patients with ST-segment elevation myocardial infarction (STEMI), inadequate myocardial reperfusion and widespread myocardial necrosis are still seen in approximately 50% of patients [129,130]. Pressure-controlled intermittent coronary sinus occlusion (PiCSO) has the potential to improve coronary microvascular function, which consists of a system of special balloons that are placed in the CS and cyclically inflate and deflate; this leads to an increase in CS pressure, helping to redistribute blood flow from the distant myocardium to the ischemic myocardium [131]. Finally, in the prematurely terminated PiCSO-AMI-I Trial, in anterior STEMI, the use of PiCSO therapy in addition to PCI did not reduce myocardial infarct size compared to conventional PCI, but it also did not cause an increase in adverse events up to 6 months [17]. PiCSO therapy in the acute phase and CS reducer devices in the chronic phase aim to improve clinical outcomes, but some studies have not yielded the desired results [17,18,132].

Prior to PiCSO as the current treatment option, various coronary venous-based treatment strategies were investigated to improve myocardial perfusion in acute ischemia. Historically, retrograde cardioplegia and coronary venous retroperfusion were developed to increase oxygen delivery to the ischemic myocardium by delivering it via the CS [133,134,135,136]. Although these techniques are no longer routinely used, they highlight the fundamental principle that modulation of coronary venous pressure can affect myocardial perfusion. The CS reducer device, specifically engineered for chronic refractory angina, features a stainless-steel mesh mounted on an hourglass-shaped balloon catheter. To accommodate the tapered anatomy of the CS, the balloon’s inflation pressure is adjustable, with diameters measuring 3 mm at the center and ranging from 7 mm to 13 mm at the distal and proximal ends. The core mechanism of the device is to create a controlled constriction within the CS via percutaneous implantation, thereby elevating coronary venous pressure [137]. When implementing strategies to increase CS-mediated CVS pressure, a crucial consideration is the critical threshold pressure value. This principle was established in a historical cornerstone study, in which Gott et al. observed that mean CS pressure exceeding 40 mm Hg during coronary retroperfusion led to myocardial ecchymosis; furthermore, they noted that systolic pressures above 80 mm Hg resulted in more extensive myocardial damage [138].

Well-designed follow-up studies on the CVS can not only positively contribute to reducing ACS-related mortality and morbidity, but may also help to improve the chances of success in interventions performed via CS by facilitating appropriate patient selection.

### 3.5. New Knowledge, Novelty, and Future Directions

The existing evidence suggests that ACS management is shifting from standardized protocols toward risk-stratified and mechanism-based approaches [139]. In this context, it is essential to conduct further research into the etiology and treatment of ACS by exploring the coronary circulation through novel perspectives. This review aims to explore the role of the CVS in ACS, offering a holistic understanding of the syndrome by encompassing its arterial, microvascular, and venous components. This scope includes the potential etiological contribution of coronary venous abnormalities to ACS, the role of venous dysfunction in MINOCA, and the implications for CS-based interventional strategies. By integrating these three vascular components into a unified framework, this review provides a more comprehensive understanding of ACS pathophysiology.

## 4. Conclusions

This literature review examined the CVS, with a specific focus on the hemodynamic effects of coronary veins and their potential etiological role in ACS. The existing evidence suggests that CSBF significantly decreases during the acute phase of ACS and subsequently increases following reperfusion. However, this evidence has been primarily derived from retrospective case–control studies with limited sample sizes. The absence of prospective randomized controlled trials remains a significant barrier to a comprehensive understanding of these mechanisms. Adopting a more holistic research approach integrating the CVS with existing studies on coronary arteries and the microvascular bed may be pivotal in reducing the mortality and morbidity associated with ACS.

## Figures and Tables

**Table 1:** Studies examining the coronary venous system in CAD, including ACS.

Authors, Year	Patients, Design	Method	Result
Mundigler et al. 1997 [97]	16 CAD patients, case–control	TEE, CFR in CS	Decrease in CFR in stenotic LAD lesion
Vrublevsky A. et al. 2004 [98]	65 CAD patients, case–control	TEE, CFR in CS	Decrease in CFR < 2.0
Ng Daniel et al. 2004 [99]	15 patients, before and after CABG	TTE, CSBF in CS	Increase in CSBF after reperfusion
Nishino et al. 2006 [100]	16 DM-MVD patients, case–control	TEE, CFR in CS	Decrease in CFR in diabetic MVD
Tabel et al. 2006 [101]	14 CAD patients, case–control	TTE, CFR in CS	Decrease in CFR in patients with CAD
Hajaghaei et al. 2007 [102]	19 patients, before and after CABG	TTE, CFR in CS	Increase in CFR after reperfusion
Toufan et al. 2007 [103]	20 Anterior AMI, case–control	TTE, CSBF, and CSVTI	Decrease in CSBF and CSVTI
Zheng et al. 2012 [104]	36 patients HT-CAD, case–control	TTE, CSBF in CS	Decrease in CSBF in hypertensive patients with CAD
Haridasan et al. 2013 [105]	41 patients with AP, case–control	CAG, CSFT in CS	CSFT delay in patients with AP
Akşit et al. 2020 [52]	13 CSF patients, case–control	CMR, CS flow in CS	Significant relationship between CSF and CS reflux
Meenakshi et al. 2013 [106]	232 CAD patients, case–control	TTE, CSBF in CS	Increase in CSBF after reperfusion
Nakamori et al. 2018 [107]	96 CAD patients, retrospective	CMR, CFR in CS	Decrease in CFR is consistent with the decrease in FFR
Lyubarova et al. 2018 [108]	24 CAD patients, case–control	TTE, CSBF in CS	CSBF increased after PCI
Indorkar et al. 2019 [109]	507 known or suspected patients with CAD, cohort	CMR, CFR in CS	CFR is an independent predictor of MACE
Sambasiva et al. 2019 [110]	50 STEMI patients, retrospective	TTE, CSBF, and CSVTI	Decrease in CSBF and CSVTI
Hamaya et al. 2020 [111]	237 STEMI patients, cohort	CMR, MBF in CS	Higher MACE risk with low hyperemic MBF
Kato et al. 2020 [112]	326 DM with known or suspected CAD patients, retrospective	CMR, CFR in CS	CFR < 2.0, higher MACE
Kato et al. 2021 [113]	693 CAD patients, retrospective	CMR, CSBF in CS	Increased MACE risk with higher CSBF
Al-Rikabi et al. 2021 [114]	92 CAD patients, case–control	TTE, CSBF in CS	Increase in CSBF after reperfusion
Kanaji et al. 2022 [115]	523 AMI patients, retrospective	CMR, CSBF, and CFR	Impaired g-CFR and h-CSF
Gyllenhammar et al. 2022 [116]	19 CAD patients, case–control	CMR, GMP in CS	Decrease in GMP in CAD
Banjanovic et al. 2022 [117]	61 patients, before and after CABG	TTE, CS flow CSVTI	Increase in CSBF after reperfusion
Nakamura et al. 2023 [118]	933 known or suspected patients with CAD, cohort	CMR, CFR in CS	CFR < 2.5, higher MACE
Abhiram et al. 2024 [119]	100 ACS patients, before and after revascularization	TTE, CSBF in CS	Decrease in CSBF in patients with ACS, increase after reperfusion
Sethi et al. 2024 [120]	50 STEMI patients, retrospective	TTE, CSBF, and CSVTI	Decrease in CSBF and CSVTI
Parvathareddy et al. 2025 [121]	70 CAD patients, case–control	TTE, CSBF in CS	Decrease in CSBF in CAD
Kanai et al. 2025 [122]	40 CAD patients, case–control	PET-derived MFR, CCTA CS start time	Delayed CS start time-linked to reduced MFR
Ikeda et al. 2025 [123]	119 CAD patients, retrospective	^13^N ammonia PET-CMR, MFR, and CFR	Moderate correlation but poor agreement MR-CFR vs. PET-MFR
Akşit et al. 2026 [124]	50 CAD patients, retrospective cohort	TTE, CSBF	Basal high CSBF linked to refractory angina, but not to long-term risk of ACS

Abbreviations: acute coronary syndrome (ACS), acute myocardial infarction (AMI), angina pectoris (AP), cardiac magnetic resonance (CMR), coronary angiography (CAG), coronary artery bypass grafting (CABG), coronary artery disease (CAD), coronary computed tomography angiography (CCTA), coronary flow reserve (CFR), coronary sinus (CS), coronary sinus blood flow (CSBF), coronary sinus filling time (CSFT), diabetes mellitus (DM), fractional flow reserve (FFR), global coronary flow reserve (g-CFR), global myocardial perfusion (GMP), hyperemic coronary sinus flow (h-CSF), hypertension, (HT), major adverse cardiac events (MACE), microvascular dysfunction (MVD), myocardial blood flow (MBF), myocardial flow reserve (MFR), percutaneous coronary intervention (PCI), positron emission tomography (PET), ST-segment elevation myocardial infarction (STEMI), transesophageal echocardiography (TEE), transthoracic echocardiography (TTE), velocity time integral (VTI).

## Data Availability

No new data were created or analyzed in this review. Data sharing is not applicable to this review.

## Abbreviations

The following abbreviations are used in this manuscript:

ACS	Acute coronary syndrome
AMI	Acute myocardial infarction
AP	Angina pectoris
CABG	Coronary artery bypass grafting
CAD	Coronary artery disease
CAG	Coronary angiography
CCTA	Coronary computed tomography angiography
CFR	Coronary flow reserve
CMR	Cardiac magnetic resonance
COUP-TFII	Chicken ovalbumin upstream promoter-transcription factor -II
CoVI	Coronary venous insufficiency
CS	Coronary sinus
CSBF	Coronary sinus blood flow
CVD	Cardiovascular disease
CVS	Coronary venous system
EPDCs	Epicardial-derived cells
FFR	Fractional flow reserve
g-CFR	Global coronary flow reserve
h-CSF	Hyperemic coronary sinus flow
LAD	Left anterior descending artery
LMCA	Left main coronary artery
LVEF	Left ventricular ejection fraction
MACE	Major adverse cardiac events
MFR	Myocardial flow reserve
MINOCA	Myocardial infarction with non-obstructive coronary arteries
MVB	Myocardial venous bridge
MVD	Microvascular dysfunction
PCI	Percutaneous coronary intervention
PDGF	Platelet-derived growth factor
PET	Positron emission tomography
PiCSO	Pressure-controlled intermittent coronary sinus occlusion
PLSVC	Persistent left superior vena cava
STEMI	ST-segment elevation myocardial infarction
TEE	Transesophageal echocardiography
TGF-β	Transforming growth factor-beta
TTE	Transthoracic echocardiography
VEGF	Vascular endothelial growth factor
VTI	Velocity time integral
